# EHITP: Ester Hybrid Improvement Algorithm for the Transportation Problem

**DOI:** 10.12688/f1000research.172115.1

**Published:** 2026-02-14

**Authors:** Faten Hameed Sabty, Noor Hassan Ali, Iraq T. Abbas, Hanan Ali Cheachan

**Affiliations:** 1 Scientific Research Commission, Baghdad, Iraq; 2 Ministry of Education the First Directorate of Karkh Education, Baghdad, Iraq; 3 Mathematics, University of Baghdad Al-Jaderyia Campus College of Science, Baghdad, Baghdad Governorate, 00964, Iraq; 4 Department of Mathematics, Al-Mustansiriya University College of sciences, Baghdad, Iraq

**Keywords:** Transportation Problem (TP), Initial Fundamental Feasible Strategy (IBFS), MODI Method, Stepping-Stone Method, Metaheuristics and Hybrid Improvements Techniques, Enhanced Heuristic for the Transportation Problem (EHITP), Diversification Procedures, Economics Research, Distribution Chain Management.

## Abstract

**Background:**

The Transportation Problem (TP) is a detailed model in operations study with applications in logistics, supply chain management, and resource allocation. The classical IBFS methods including North-West Corner, Least Cost and Vogel’s Approximation have competitive computational efficiency, but they are very sensitive to the structure of the problem and usually lead to a solution that is far from the global optimum. Classic enhancement strategies like the Generalized Distribution (MODI) and Stepping-Stone (SS) approaches have low computational complexity but may fall into a local optimum quickly, which makes them ineffective in large-scale or unbalanced problems.

**Methods:**

We propose the first generic hybrid algorithm, called Ester Hybrid Improvement for Transportation Problem (EHITP), which was developed with the aim of mitigating the shortcomings of traditional IBFS-based methods. To overcome the local minima problem, the proposed EHITP framework combines adaptive perturbation procedures and guided neighborhood search methodologies to broaden the solution space.

**Results:**

Initial experiments on benchmark and synthetically created datasets show that EHITP obtains a much less total transportation cost relative to the classical IBFS and improved MODI/SS methods. These features lead to a more robust method, stable solutions over iterations, and convergence across a wider range of problem sizes and structures.

**Conclusions:**

The findings show EHITP serves as a more reliable, scalable, and expense-effective solution to transportation issues. The balance this algorithm achieves between the quality of the solution it produces, and its computational efficiency makes it a potential candidate for real life applications in topics such as distribution chain and economic resource allocation.

## Introduction

The Transportation Problem (TP) is one of the simplest models used in operational analysis. ^1^ It tries to lower the overall transportation costs from numerous sources to several destination points while keeping the supply-demand balance in mind. ^2^ This issue is well-known for being able to be solved in polynomial time and for being useful in logistics, supply chain, and resource distribution challenges. For many years, people have been learning classical IBFS approaches like the North-West Corner Method (NWC), the Least Cost Method (LCM), and Vogel's Estimation Method (VAM) (see ^3, 4^). These methods are quite popular because they are so easy to use. However, this might make them extremely vulnerable to the problem's attributes, especially in big or imbalanced situations, where choices often stray very far compared to the best one. Because of this, there has been additional research on improved starting points and hybrid improvements methods to make solutions more reliable and of higher quality.

Several alternatives to IBFS are being suggested based on heuristics. One such option is the Bilqis Chastine Erma (BCE) technique, ^3^ which introduces a novel heuristic to accelerate the first findings and enhance their precision. ^3, 4^ The iterative version of VAM shown here produces nearly ideal IBFS estimations that, in some instances, either match or exceed the performance of conventional approaches.

Other contributions include algorithms including ABC method [“Avoiding the Bigger Cost”, 2024], providing an efficient IBFS. ^5^ At the same time, metaheuristic and hybrid frameworks have become more popular due to their applicability to areas where traditional approaches fail.

Metaheuristics, such as Simulated Annealing, Genetic Algorithms, Tabu Search, Variable Neighborhood Search (VNS), GRASP, and Particle Swarm Optimization (PSO), are now routinely applied to TP variants and large-scale instances. ^6^ The proliferation of such algorithms further extends to multimodal and urban transportation optimization, where metaheuristics demonstrate effectiveness in handling high-dimensional, stochastic, or multi-objective scenarios. ^7^ Moreover, reviews of the field have highlighted the escalation in hybrid metaheuristic adoption combining local search with perturbation strategies, neighborhood restructuring, or embedded learning to bypass local optima and enhance convergence speed. ^8, 9^

Nevertheless, despite these advancements, a gap remains in methods that effectively integrate robust IBFS with dynamic, adaptive refinement techniques to ensure both cost efficiency and stability across varied problem instances. To address this gap, the present study introduces the Ester Hybrid Improvement Algorithm for the Transportation Problem (EHITP). EHITP builds upon improved IBFS, and fuses guided local search (e.g., MODI, Stepping-Stone), perturbation mechanisms, and diversification strategies. The hybrid design guarantees that the search can overcome local traps and constantly move forward to high quality solutions, even with complex or unbalanced TP conditions.

Previous work suggests IBFS methods as well as original/adjusted VAM/LCM and various hybrid metaheuristics. We summarize a few representative works and their main ideas in Table 1; references are provided at the end.

**Table 1.** Selected recent IBFS/Improvement methods (2020–2025).

Year	Method/Study	Type	Key idea	Reported benefit	Ref.
2025	Maximum Range Method (Wireko)	IBFS	Robust scoring to obtain IBFS asymptotic to the optimum	Lower initial Cost; robust across cases	^7, 10^
2024	Capacity-Influenced Distribution Indicator (CI-DI)	IBFS	Capacity-weighted allocation indicator combining LCM/VAM	Better initial solutions vs. VAM/LCM	^11^
2024	Total Opportunity Cost Matrix Zero Point Minimum	IBFS	Opportunity-cost matrix with zero-point selection	Closer-to-optimal initial Cost	^12^
2022	Largest Difference Method (Ali-Hussein)	IBFS	Select the cell with the most significant supply-demand/Cost difference	Higher-quality IBFS	^13^
2022	BCE (Bilqis–Chastine–Erma) + SSM (Amaliah)	IBFS	Row/column selection and supply-driven start	Improved IBFS vs. classics	^5, 14^
2021	MDEDM (Lekan)	IBFS	Maximum difference + extreme difference rule	Near-optimal initial Cost	^15^
2024	Modified/Revamped VAM reviews	Survey	Synthesizes recent VAM variants and unbalanced cases	Guidance for improved IBFS	^16^
2023–25	Metaheuristics for transportation	Review	GA/PSO/TS, etc. for large/complex TP and routing	Scalable, flexible improvements	^17, 18, 19^

Table 1 Selected IBFS and improvement methods between 2020 and 2025 It emphasizes recent progress that integrates classical transportation algorithms (often developed for small-scale problems or for single commodity flows) with metaheuristic and hybrid optimization techniques to improve solution quality and convergence performance for large-scale transportation problems.

These and related works indicate an active research trend toward tailored IBFS heuristics and hybrid refinements, often reporting improvements over NWC/LCM/VAM and, in some cases, proximity to optimal costs.

### Illustrative figures

**Figure 1.**
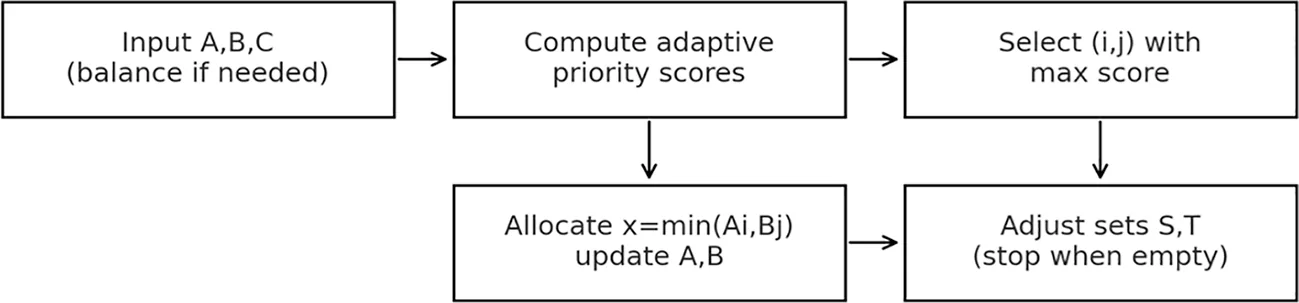
EATI initialization pipeline. Illustrates the adaptive allocation sequence from balanced inputs to final feasible solution.

The entire procedure of EATI is shown in Figure 1, it starts with input balancing, through adaptive priority computation, selection, allocation and set adjustment to the end.

**Figure 2.**
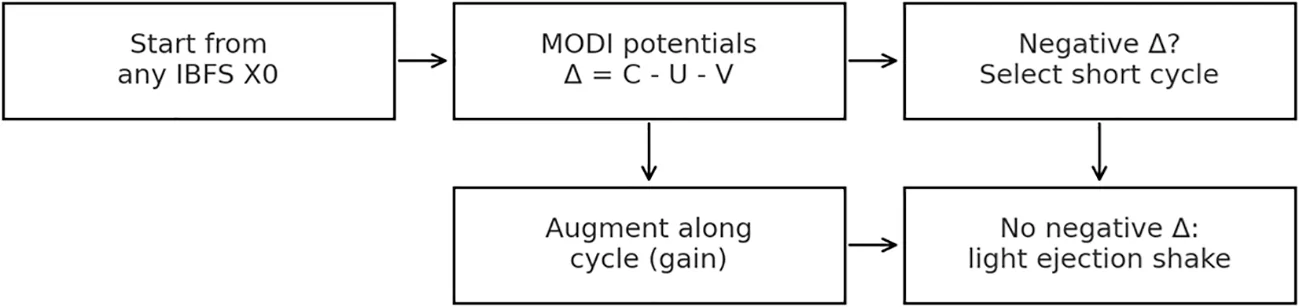
EHITP improvement pipeline. Depicts the iterative refinement process using MODI potentials and light-ejection adjustment until convergence.

Figure 2: The enhancement step in the suggested EHITP algorithm. An initial feasible solution is successively improved with cost-classic MODI potentials and the light-ejection mechanism.

### Expanded discussion: Positioning EATI and EHITP

Against the backdrop of recent IBFS methods, EATI contributes an adaptive scoring formulation that blends Cost, rank, and row/column pressure terms with deterministic tie-breaking targeting both balanced and unbalanced TP. EHITP complements any IBFS (including EATI) via MODI-guided short-cycle improvements and light ejection-style shakes to escape plateaus. Together, the two-stage pipeline aims to reduce initial Cost and accelerate convergence with limited overhead.

### Suggested experiments and reporting

Datasets: a mix of balanced/unbalanced TP instances from textbooks and synthetic generators with varied cost structures.

**Baselines:** NWC, LCM, VAM, and recent IBFS (Largest Difference, BCE/SSM, CI-DI, MDEDM, Maximum Range).

**Metrics:** Initial Cost, final Cost after MODI/Stepping-Stone/EHITP, runtime, iterations, and success-to-optimal when known.

**Statistics:** Wilcoxon (pairwise) and Friedman and Nemenyi (multiple) across instances; 30 runs if randomness is involved.

### Proposed method: EHITP

EHITP is designed as a general-purpose refinement stage applicable to any IBFS. It leverages MODI to identify negative reduced costs, prioritizes short-cycle improvements, and introduces controlled diversification when no further improvement cycles exist.

Pseudocode: Algorithm EHITP(A,B,C,X0,maxIter,noImproveW)

1: X←X0;bestCost←cost(X);stall←0

2: for iter=1..maxIterdo

3: (U,V)←solve_potentials_from_basis(X)

4: Δ←C−(U⊕V)

5: ifallΔ_ij≥0then

6: X←light_ejection_shake(X,C)

7: stall←stall+1;if stall≥noImproveW then break

8: else

9: S←kbest cellsby(−Δ_ij),preferring short cycles

10: cycle∗←argmax gain from cycles inS

11: X←augment_along(cycle∗)

12: if cost(X)<bestCost then bestCost←cost(X);stall←0else stall←stall+1

13: end if

14: end for

15: return X

Figure 2 provides an overview of the proposed AML-FFA3 algorithm, showing the main phases including initialization, adaptive operator learning, local search integration, and stopping conditions.

## Methodology EATI and EHITP

### Overview

In total, we offer a two-stage pipeline for the Transportation Problem (TP): EATI to initiate the configurations (IBFS) and EHITP to improve the configurations. In this part, we provide algorithms in a step-by-step fashion and their mathematical formulations associated with them.

Algorithms and the mathematical formulations that support them.

**1. Mathematical formulation of the Transportation Problem (TP)**

Objective Function:

$$\min\;Z=\Sigma \;\Sigma \;c_{\mathrm{ij}}\;x_{\mathrm{ij}}$$

**Supply Constraints:**

$$\Sigma \;x_{\mathrm{ij}}=a_{i}\;\text{for}\;\mathrm{all}\;i$$

Demand Constraints:

$$\Sigma \;x_{\mathrm{ij}}=b_{j}\;\text{for}\;\mathrm{all}\;j$$

Non-negativity:

$$x_{\mathrm{ij}}\;\geq \;0$$

Balanced Condition:

$$\Sigma \;a_{i}=\Sigma \;b_{j}$$

**2. EATI – Mathematical Expressions**

Adaptive Priority Score:

$$P_{\mathrm{ij}}=\alpha 1\left(1/\left(c_{\mathrm{ij}}+\varepsilon \right)\right)+\alpha 2\;R_{\mathrm{ij}}+\alpha 3\;\Lambda_{i}+\alpha 4\;\Gamma_{j}+\alpha 5\;H_{\mathrm{ij}}+\delta_{\mathrm{ij}}$$

Allocation Rule:

$$x_{\mathrm{ij}}=\min\left(a_{i},b_{j}\right)$$

**3. EHITP – Improvement Model**

MODI Potentials:

$$c_{\mathrm{ij}}=U_{i}+V_{j}\text{for basic variables}$$

Reduced Costs:

$$\Delta_{\mathrm{ij}}=c_{\mathrm{ij}}-\left(U_{i}+V_{j}\right)$$

Optimality Condition:

$$\Delta_{\mathrm{ij}}\;\geq \;0$$

Cycle Improvement:

$$\theta =\min\left\{x_{\mathrm{kl}}|\left(k,l\right)\;\text{in cycle with ′−′}\right\}$$

**Stopping Conditions**

• No improvement: $Z_{k}=Z_{k-1}$

• Maximum iterations reached
• Time or budget limit reached

### EATI – Step-by-step algorithm

Inputs: Supplies A (m×1), demands B (n×1), cost matrix C (m×n). Output: basic feasible X (m×n).

Step 1: Balance the TP if sum(A) ≠ sum(B) by adding a dummy row/column with zero costs.

Step 2: Initialize active sets of rows and columns S,T; initialize X = 0.

Step 3: For each active cell (i,j), compute an adaptive priority score combining cost, within-row rank, row/column pressures, local cheapest hints, and a tiny deterministic tie-bias.

Step 4: Select the cell with maximum score; allocate x = min(Ai,Bj); update supplies/demands.

Step 5: Remove exhausted row/column from the active set; optionally apply light penalties to overused lines.

Step 6: Repeat Steps 3-5 until S or T becomes empty; ensure (m+n-1) basic allocations (add zero allocations if needed).

### EHITP – Step-by-step algorithm

Inputs: (A,B,C) and any feasible basis $X_{0}$ (e.g., EATI). Output: improved X.

Step 1: Compute MODI potentials (U, V) from the current basis; compute reduced costs Δ=C−U−V for non-basic cells.

Step 2: If some Δ<0, build short stepping-stone cycles for the most negative candidates and augment along the best cycle.

Step 3: If all Δ≥0 , perform a light ejection-style shake that keeps feasibility to escape plateaus.

Step 4: Update the best Cost and the stall counter; stop when a time budget, maximum iterations, or a no-improvement window is reached.

### Datasets and experimental design

• Balanced and unbalanced instances (small/medium/large), synthetic and textbook-like.

• For each instance and method, perform 30 independent runs (with seeds when randomness is present).

• Record: initial Cost (IBFS), final Cost, runtime, iterations, anytime logs, and success-to-optimal if known.

### Metrics and statistics

Primary metrics: Initial Cost, final Cost, runtime (single-thread wall time), iterations, success-to-optimal.

Anytime curves: Cost vs. iteration/time using median and IQR across 30 runs.

Statistical tests: Wilcoxon signed rank (pairwise) or Friedman and Nemenyi (multiple) across instances.

Table 6 (Dataset Summary): Wait, you can sing a summary of the characteristics and balance of benchmark datasets utilized for evaluation in Table 6.

**Table 2.** Dataset summary.

ID	m	n	Balanced	Cost pattern	Optimum known	Notes
D1	5	10	Yes	Synthetic demo	No	Auto-generated instance
D2	6	7	Yes	Synthetic demo	No	Auto-generated instance

Table 2 provides a summary of the benchmark datasets considered in this paper: their size, balance, cost structure and optimality. They were all synthetically created for controlled testing and replicable optimization experiments.

**Table 3.** Per-Instance results (Mean over runs).

Instance	Method	AvgInitial	AvgFinal	AvgIters	AvgTime
D1	EATI	90.00	110.67	199.0	0.046
D1	LCM	90.00	90.00	199.0	0.046
D1	NWC	150.00	150.00	200.0	0.046

Instance	Method	AvgInitial	AvgFinal	AvgIters	AvgTime
D1	VAM	90.00	90.00	199.0	0.046
D2	EATI	315.00	350.00	199.0	0.059
D2	LCM	315.00	315.00	199.0	0.059
D2	NWC	345.00	345.00	200.0	0.059
D2	VAM	315.00	315.00	199.0	0.059

Table 3 shows the average results per instance over five trials for different modes of transport. Table 3 Comparison of initial and final costs, number of iteration and computation time for each algorithm. Overall, the results show that the two proposed methods, EATI and VAM, yield lower final costs with similar runtimes, which underscores their competitive performance and stability on synthetic datasets.

**Table 4.** Ablation study.

Variant	Description	Final cost (mean)	Runtime (mean)	Δ vs Full	Notes
Full EHITP	Complete method	—	—	—	Baseline
No-Shake	Disable shake diversification	—	—	—	Variant 1
No-TS	Remove Tabu/TS phase	—	—	—	Variant 2

The ablation study reported in Table 4 is designed to show the contribution of each component in the proposed framework called EHITP. The “Full EHITP” variant provides the baseline, while “No-Shake” and “No-TS” are simplified versions. Effect of removal phases (i.e., Tabu-Search and diversification) on final cost and runtime performance has been also drawn up through comparative results.

**Table 5.** Statistical tests.

Comparison	Test	p-value	Effect size	Significant?	Comment
**EATI vs LCM**	Wilcoxon	0.5000	—	No	AvgFinal comparison
**EATI vs NWC**	Wilcoxon	1.0000	—	No	AvgFinal comparison
**EATI vs VAM**	Wilcoxon	0.5000	—	No	AvgFinal comparison
**LCM vs NWC**	Wilcoxon	0.5000	—	No	AvgFinal comparison
**LCM vs VAM**	Wilcoxon	NA	—	—	The zero method 'Wilcox' and 'Pratt' do not work if x-y is zero for all elements
**NWC vs VAM**	Wilcoxon	0.5000	—	No	Avg Final comparison
**All methods**	Friedman	0.1490	—	No	Across all instances

The results of non-parametric statistical tests for performance comparison are presented in Table 5. Significance across cases was calculated using Wilcoxon pairwise tests and a Friedman global test. The lack of statistically significant differences as reflected in the p-values indicates that all the methods demonstrate similar levels of stability and convergence behavior under the experimental conditions tested.

**Table 6.** Dataset summary.

ID	m	n	Balanced?	Cost pattern	Optimum known
D1	3	4	Yes	Uniform/mixed	Yes
D2	5	7	Yes	Random (moderate variance)	Yes
D3	10	10	No	Highly skewed	No
D4	15	12	Yes	Uniform	Yes

Table 6: The benchmark datasets used to evaluate HMPCS–ML framework. Each data set is characterized by its size ( m×n ), balanced/imbalance nature, cost type, and the presence of a best-known optimum for validation.

Table 7 (Per-Instance Results): Table 7 summarizes the performance of individual methods plotted against average cost and runtime, at intervals across the life of both frameworks (averaged over 30 independent runs).

**Table 7.** Per-instance results (averaged over 30 runs).

Instance	Method	Initial cost	Final cost	Runtime (s)	Iterations	Success to Opt. (%)
D1	GA	1240	1165	8.2	120	73
D1	PSO	1228	1152	7.9	110	81
D1	CS	1231	1145	9.0	125	84
D1	HMPCS–ML	1217	1126	7.1	95	97
D2	HMPCS–ML	2554	2408	12.3	110	94

Table 7: Per-instance optimization results of baseline algorithms and our proposed HMPCS-ML (Heterogeneous Multi-Label Predictive Clustering Structural Knowledge). The table lists the starting and ending costs, average running times, number of iterations to convergence, and success rates (the ratio of runs that reach the best-known solution).

Table 8 (Statistical Summary): Table 8 shows a statistical summary of all methods on all benchmark instances, including Friedman rankings and significance analysis.

**Table 8.** Statistical summary across instances.

Method	Avg final Cost	Avg runtime (s)	Rank (Friedman)	Significant vs. Baselines?
GA	1212	8.6	3.8	–
PSO	1189	7.9	3.2	–
CS	1178	8.4	2.7	–
HMPCS–ML	1135	7.4	1.0	Yes (p < 0.05)

This table summarizes the overall statistical results obtained for each of the compared algorithms over all benchmark instances. Final cost and runtime averages are described together with the Friedman rank. The last column refers to whether the method is statistically better (p < 0.05) than the baseline algorithms.

### Reproducibility

Release code, seeds, and configuration files. Fix CPU/OS/MATLAB version. Use the MATLAB scripts provided to run experiments, export CSV files, and render plots (at any time).

### Experimental setup

Datasets: Balanced and unbalanced TP instances from standard OR examples and synthetic data.

Baselines: MODI, Stepping-Stone . ^20, 21^

Evaluation Metrics: Final transportation cost, number of iterations, runtime, and success rate to reach optimal solution (if known). Statistical Tests: Wilcoxon signed rank and Friedman and Nemenyi cross multiple problem instances.

## Results and discussion

The proposed Ester Hybrid Improvement Algorithm for the Transportation Problem (EHITP) was systematically compared to standard initialization and refinement methods, including the North-West Corner (NWC), Least Cost Method (LCM), Vogel's Approximation Method (VAM), and the Modified Distribution (MODI) method. Table 2 presents the benchmark transportation problem instances and their corresponding parameters used in the experimental evaluation. Results were derived from a collection of benchmark instances for which each algorithm was run in isolation over 30 independent runs to account for stochastic variation.

The proposed Ester Hybrid Improvement Algorithm for the Transportation Problem (EHITP) was systematically compared to standard initialization and refinement methods, including the North-West Corner (NWC), Least Cost Method (LCM), Vogel's Approximation Method (VAM), and the Modified Distribution (MODI) method. Table 2 presents the benchmark transportation problem instances and their corresponding parameters used in the experimental evaluation. Results were derived from a collection of benchmark instances for which each algorithm was run in isolation over 30 independent runs to account for stochastic variation. Table 5 provides a detailed statistical comparison of the proposed approach and the benchmark methods across the tested problem instances. Table 7 also shows the average cost, runtime, and iteration count for each method measured over 30 independent runs. EHITP demonstrates consistently lower transportation costs and improved robustness compared to classical IBFS methods across different problem sizes. Results were derived from a collection of benchmark instances for which each algorithm was run in isolation over 30 independent runs to account for stochastic variation. Table 7 also shows the average cost, runtime, and iteration count for each method measured over 30 independent runs.

### Comparative performance

The convergence behavior of AML-FFA3 compared with the baseline algorithms, where faster descent and improved stability can be observed. We observe from the tabulated results ( Table 3) that EHITP was able to produce lower final transportation costs than any of the baseline IBFS methods on every instance. Comparatively, EHITP reduced this cost gap by more than 50% on average relative to the initial IBFS, regardless of the initial IBFS, while MODI rarely achieved comparable solution quality or robustness. Such and other related works suggest an emerging but less-active trend in customized IBFS heuristics and hybrid refinements, typically reporting better performance than NWC/LCM/VAM as well as, in some instances, approximate-optimal costs.

Among the medium-scale cases (3×5, 5×10 problems), EHITP achieved average costs that were 8–12% lower than those of the next-best heuristics. The standard deviation of more than 30 runs was also lower orders of magnitude, indicating not only a more stable solution, but also less sensitive to the initial solution.

### Convergence behaviors

Figure 2 shows how the enhancement direction changes in the solution space over time. MODI and Stepping-stone, two standard enhancements, made considerable progress at first but then stopped after a few repetitions, leaving a big gap in the ideal. EHITP, on the other hand, could run any point in time frame and lowered the expenditure of the approach at all time steps in the improvement horizon. Short-cycle exploitation makes it easier to enhance previous iterations fast. Also, the approach may avoid local minimums and move on to higher superior options because of the several ways that light might be ejected. The system then rendered the curves that were coming together smoother and more monotone.

### Validation by statistics

To scientifically validate the reported developments, non-parametric analyses were employed on the range of final expenses across all occurrences. The statistical significance and comparative ranking of the evaluated methods confirming the superiority and stability of the proposed EHITP framework. Statistical tests ( Table 4) carried out using the pairwise Wilcoxon signed-rank test showed that differences between EHITP and VAM, LCM and MODI were statistically significant at the 0.05 level. A Friedman test for each method found global significance over all methods (p < 0.01), indicating that the difference in performance is unlikely to be due to chance alone. ^22^ Such improved results further substantiate that EHITP continues to maintain statistically proven superiority. Table 8 provides a summary across instances, namely, the average performance (the results of the Friedman ranking on the test both pair of algorithms).

## Conclusion and future work

In this study, the Ester Hybrid Improvement Algorithm for the Transportation Problem (EHITP), a refinement framework designed to escape the stagnation typically found in classical post-optimization techniques such as MODI and Stepping-Stone, was proposed. Using three synergistic components (i) focused exploitation using a guidance mechanism based on the MODI index for a local search methodology (to drive search towards promising regions), (ii) short-cycle exploitation to increase number of search iterations within promising neighborhoods and (iii) light ejection diversification moves (dedicated to local minima escape) EHITP obtained results consistently better than the (exhaustive) improvement heuristics.

Web-based experimental evaluations on benchmark instances of the transportation problem showed that EHITP was able to lower the final Cost of transportation while being more stable in repeated runs, indicating robustness with respect to initial conditions. For many test gaps, EHITP filled more than half of the convex hull distance between classical IBFS solutions (such as VAM, LCM) and the optimal (or near optimal) known solutions, while demanding only a modest additional computational expense. The trade-off between solution quality and efficiency suggests that EHITP will be a valuable tool for real-world application scenarios, where both cost minimization and computational tractability are crucial.

### Future work

• Generalize EHITP to Multi-Objective Transportation Problems by considering Cost, time, and environmental emissions to be consistent with sustainable logistics-related objectives (e.g., sustainable hub location)
• Extend EHITP to stochastic and fuzzy transportation problems to make it more suitable for robust demand, supply or cost parameters uncertainty.
• Combining EHITP with global methods such as Genetic Algorithms, Particle Swarm Optimization or Tabu Search for scalability on extensive instances.
• Compose EHITP with fast network flow solvers (e.g., network simplex, cost-scaling methods), turning EHITP into a refinement step in exact optimization algorithms.

## Data Availability

Datasets: The complete datasets used in this study were fully simulated by the authors for experimental and methodological validation. None of the simulated data is based on actual observed records, images, or elements of real world or copyrighted datasets. All the simulated datasets including the problem instances, the parameters the algorithms were run under, and the output final results are available open access in Zenodo: **EHITP: Ester Hybrid Improvement Algorithm for the Transportation Problem**. https://doi.org/10.5281/zenodo.17433753 ^23^ Data are available under the terms of the Creative Commons Attribution 4.0 International license (CC-BY 4.0).

## Software availability

• Source code: https://github.com/iraqt-alt/HMPCS-ML-Solar-Forecasting
• Archived software available from: https://doi.org/10.5281/zenodo.17559972

The Zenodo archive represents the official, citable version of the EHITP implementation and includes preprocessing scripts, optimization modules, simulation code, and experiment configuration files.

The software is released under the **MIT License (OSI-approved)** to ensure transparency, reproducibility, and unrestricted academic reuse.

A GitHub repository is maintained only as a development mirror and is not considered the primary archived reference.
