# Supplementary material for: EHITP: Ester Hybrid Improvement Algorithm for the Transportation Problem
Source: F1000Res. 2026 Apr 24;15:263. Originally published 2026 Feb 14. [Version 2] doi: 10.12688/f1000research.172115.2 (PMC13555067; doi:10.12688/f1000research.172115.2)
Supplement: Peer review history

# Reviewer report: Reviewer response for version 2

**DOI:** 10.5256/f1000research.198586.r499250

**Published:** 2026-07-21

**Authors:** Mushtak A.K. Shiker

**Affiliations:** 1 Mathematics, University of Babylon, Hilla, Babylon Governorate, Iraq

This research paper proposes a valuable update to one of the methods for finding solutions to transportation problems. I commend the quality of the work, but the following points should be considered:

 1. Linguistic revision is needed to avoid errors.

 2. Recent sources relevant to the core topic should be added.

 3. A comparison should be made between the proposed method and classical methods, along with some recent improvements.

 4. Some basic mathematical terms related to the research topic should be added.

 5. Some duplicate results should be removed, and the scope, significance, and findings of the work should be highlighted.

 6. The references should be rewritten and standardized according to established scientific conventions.

 7. Discuss the tables and figures in more detail.

 8. Discuss the results of the comparisons in detail, highlighting the similarities and differences.

Is the work clearly and accurately presented and does it cite the current literature?

Yes

If applicable, is the statistical analysis and its interpretation appropriate?

Yes

Are all the source data underlying the results available to ensure full reproducibility?

Yes

Is the study design appropriate and is the work technically sound?

Yes

Are the conclusions drawn adequately supported by the results?

Yes

Are sufficient details of methods and analysis provided to allow replication by others?

Yes

Reviewer Expertise:

Operation Research ; Optimization ; Applied Mathematics ; Linear Systems of Equations ; Non-Linear Systems of Equations .

I confirm that I have read this submission and believe that I have an appropriate level of expertise to confirm that it is of an acceptable scientific standard.

## References

[1] : A Modification to Vogel’s Approximation Method to Solve Transportation Problems. *Journal of Physics: Conference Series* .2020;1591(1) : 10.1088/1742-6596/1591/1/012029 10.1088/1742-6596/1591/1/012029

[2] : A New Revised Efficient of VAM to Find the Initial Solution for the Transportation Problem. *Journal of Physics: Conference Series* .2020;1591(1) : 10.1088/1742-6596/1591/1/012032 10.1088/1742-6596/1591/1/012032

# Reviewer report: Reviewer response for version 2

**DOI:** 10.5256/f1000research.198586.r499252

**Published:** 2026-07-16

**Authors:** Israa H. Hasan

**Affiliations:** 1 University of Technology, Baghdad, Iraq

**General Summary**

 A well-written paper proposing a practical hybrid algorithm (EHITP) for logistics optimization. The results are solid, but some scientific and language edits are required to enhance its quality.

 ​ **Scientific Remarks:**

 ​ **Novelty Demonstration:** The novelty of the proposed EHITP algorithm should be further demonstrated by comparing it to the closest relevant hybrid transportation optimization methods.

 ​ **Case Studies:** It is suggested that additional reference transportation cases with varying sizes and characteristics be added.

 ​ **Mathematical Notation:** All mathematical symbols and variables should be clearly defined in this paper.

 ​ **Complexity Analysis:** To significantly enhance the research, a brief computational complexity analysis should be conducted by comparing the EHITP algorithm with fundamental methods.

 ​ **Software & Code Consistency:** The redundancy of the software should be thoroughly checked to ensure consistency between the program, the GitHub repository, and the proposed algorithm.

 ​ **Formatting and Language Remarks:**

 ​ **Structure:** Duplicate paragraphs in the Results and Discussion sections should be removed. The actual results should be reported after comparing the EHITP method with established methods.

 ​ **Grammar:** Proofreading is recommended to improve grammar.

 ​ **References:** Ensure all sources are formatted according to the journal's guidelines.

Is the work clearly and accurately presented and does it cite the current literature?

Yes

If applicable, is the statistical analysis and its interpretation appropriate?

Partly

Are all the source data underlying the results available to ensure full reproducibility?

No source data required

Is the study design appropriate and is the work technically sound?

Yes

Are the conclusions drawn adequately supported by the results?

Yes

Are sufficient details of methods and analysis provided to allow replication by others?

Yes

Reviewer Expertise:

Operation research, optimization, hybrid algorithms

I confirm that I have read this submission and believe that I have an appropriate level of expertise to confirm that it is of an acceptable scientific standard, however I have significant reservations, as outlined above.

# Reviewer report: Reviewer response for version 1

**DOI:** 10.5256/f1000research.189810.r469145

**Published:** 2026-04-06

**Authors:** Annisa Kesy Garside

**Affiliations:** 1 Universitas Muhammadiyah Malang, Malang, Indonesia

This manuscript introduces the Ester Hybrid Improvement (EHITP) algorithm which is designed to optimize the solution to the Transportation Problem (Transportation Problem - TP). The author aims to bridge the gap between the classic Initial Basic Feasible Solution (IBFS) method and the optimal solution through heuristic hybridization. This paper evaluates the performance of the algorithm compared to traditional methods such as the North-West Corner (NWC), Least Cost Method (LCM), and Vogel's Approximation Method (VAM) using small-scale numerical examples and statistical testing.

**Reviewer's Main Comment**

 Although this topic is relevant in the field of operational research, the current version of the manuscript contains several fundamental weaknesses, critical inconsistencies, and technical inaccuracies that make it not scientifically eligible.

**Specific Criticism and Necessary Revision **

1. Critical Inconsistencies in Presentation and Accuracy This manuscript has a very serious internal contradiction, which shows a lack of precision in the compilation:

- Algorithm Naming: In the results section, especially on Table 7 (page 9), the author presents the results for an algorithm named "HMPCS-ML", even though the entire paper discusses "EHITP".
- Image Reference Error: In the methodology section (page 5), the text refers to Image 2 as "AML-FFA3 improvement pipeline", but the description (caption) on Image 2 is written "EHITP improvement pipeline".Recommendation: The author should conduct a comprehensive audit of the manuscript to ensure that the algorithm explained in the methodology is the same algorithm used in the experiment. All references to "HMPCS-ML" and "AML-FFA3" should be corrected or clarified.

1. Data Availability and Reproducibility

The "Data Availability" section includes a GitHub link that should contain the EHITP algorithm code. However, the repository points to a project called "HMPCS-ML-Solar-Forecasting".

- Issue: The code in the repository is related to solar energy prediction and has no relation to the Transportation Problem or the EHITP algorithm discussed in this paper.
- Recommendation: To comply with F1000Research's open data policy, the author must provide the correct source code link and include the specific dataset (matrix) used in this research

1. Statistical Analysis and Interpretation

Mathematically incorrect interpretation of statistical results:

- Contradiction: In Table 5, the results of the Wilcoxon Signed-Rank test show that the p-value ranges from 0.5000 to 1.0000. Statistically, this value shows that there is no significant difference between EHITP and the comparative method. However, the authors conclude in the text that EHITP is "significantly superior."
- Recommendation: The authors should reevaluate their statistical tests. If the improvement in results is not statistically significant, then claims of superiority should be reduced or supported by testing on a much larger and diverse dataset to find real significance.

1. Literature Review and State of the Art (SOTA)

This paper claims to provide modern improvements, but the majority of comparisons are made against very old methods (NWC, VAM, LCM).

- Lack of Modern Context: To show real "improvements", the authors should compare their methods with contemporary hybrid algorithms or metaheuristics (for example, modified Genetic Algorithm or Particle Swarm Optimization for TP) published in the last 5 years.

 

1. Technical Details and Methodological

- Methodology: Some of the functions mentioned in the algorithm (such as light_ejection_shake) do not provide sufficient mathematical or logical explanations, so that it is difficult for the reader to fully understand the "hybrid" mechanism.
- Scalability: Experiments are limited to very small matrices. Authors should include at least one large-scale benchmark problem to prove the algorithm's efficiency in real-world scenarios.

**Conclusion and Revision Mandatory Points**

 In order for this article to be considered scientifically worthy, the following points must be improved:

1. Fix all naming inconsistencies (EHITP vs HMPCS-ML vs AML-FFA3).
2. Provide a link to the correct GitHub repository containing the specific code for the EHITP algorithm and the dataset used.
3. Align conclusions with statistical data; if the p-value is high, the claim of "statistical significance" should be removed or the test should be extended.
4. Explain the details of the sub-functions of the algorithm to allow full transparency and replication by other researchers

Is the work clearly and accurately presented and does it cite the current literature?

No

If applicable, is the statistical analysis and its interpretation appropriate?

No

Are all the source data underlying the results available to ensure full reproducibility?

No

Is the study design appropriate and is the work technically sound?

No

Are the conclusions drawn adequately supported by the results?

No

Are sufficient details of methods and analysis provided to allow replication by others?

No

Reviewer Expertise:

Operations Research, Optimization Algorithms, Logistics, and Supply Chain Management.

I confirm that I have read this submission and believe that I have an appropriate level of expertise to state that I do not consider it to be of an acceptable scientific standard, for reasons outlined above.

# Response

**Published:** 2026-04-07

**Authors:** iraq abbas

**Affiliations:** Mathematics, University of Baghdad Al-Jaderyia Campus College of Science, Baghdad, Baghdad Governorate, Iraq

Dear Editor and Reviewers,

 Thank you for your valuable comments and constructive feedback.

 We have carefully revised the manuscript to address all the concerns raised. In particular, we have corrected inconsistencies in the methodology and results sections, unified the terminology throughout the paper, and improved the statistical interpretation of the results. Additionally, the experimental tables and dataset descriptions have been updated to ensure clarity and consistency.

 We believe that the revised version of the manuscript significantly improves its scientific quality and presentation.

 We appreciate your time and consideration.

 Sincerely,  

 Iraq T. Abbas

# Reviewer report: Reviewer response for version 1

**DOI:** 10.5256/f1000research.189810.r459029

**Published:** 2026-02-23

**Authors:** Hussam Abid Ali Mohammed

**Affiliations:** 1 University of Kerbala, Karbala, Karbala Governorate, Iraq

The study presents a robust hybrid framework Enhanced Heuristic for the Transportation Problem (EHITP) that effectively integrates adaptive IBFS initialization with MODI-guided local search and diversification strategies. It demonstrates improved solution quality, enhanced stability across runs, and statistically validated performance, while maintaining computational efficiency and reproducibility through open-access datasets and source code availability.

Is the work clearly and accurately presented and does it cite the current literature?

Yes

If applicable, is the statistical analysis and its interpretation appropriate?

Partly

Are all the source data underlying the results available to ensure full reproducibility?

Yes

Is the study design appropriate and is the work technically sound?

Yes

Are the conclusions drawn adequately supported by the results?

Yes

Are sufficient details of methods and analysis provided to allow replication by others?

Yes

Reviewer Expertise:

Operation Research

I confirm that I have read this submission and believe that I have an appropriate level of expertise to confirm that it is of an acceptable scientific standard.

# Response

**Published:** 2026-04-24

**Authors:** iraq abbas

**Affiliations:** Mathematics, University of Baghdad Al-Jaderyia Campus College of Science, Baghdad, Baghdad Governorate, Iraq

Dear Reviewer,

 Thank you very much for your valuable and constructive feedback. We sincerely appreciate your positive evaluation of our work and your recognition of its contribution.

 Regarding the statistical analysis, we have carefully reviewed this aspect and made additional clarifications and minor improvements in the revised version to further strengthen the presentation and interpretation of the results.

 We are grateful for your insightful comments, which helped us improve the quality of the manuscript.

 Sincerely,

 Iraq T. Abbas
